# Variations in breast cancer surgical treatment and timing: determinants and disparities

**DOI:** 10.1007/s10549-021-06155-1

**Published:** 2021-03-10

**Authors:** Irene Dankwa-Mullan, Judy George, M. Christopher Roebuck, Joseph Tkacz, Van C Willis, Fredy Reyes, Yull E. Arriaga

**Affiliations:** 1 IBM Watson Health, 75 Binney Street, Cambridge, MA 02142 USA; 2 RxEconomics LLC, Hunt Valley, MD USA

**Keywords:** Breast cancer, Breast conserving therapy, Health disparities, Health equity, Health services research, Non-metastatic invasive breast cancer

## Abstract

**Purpose:**

To describe clinical and non-clinical factors associated with receipt of breast conserving surgery (BCS) versus mastectomy and time to surgical intervention.

**Methods:**

Cross-sectional retrospective study of January 1, 2012 through March 31, 2018 data from the IBM MarketScan Commercial Claims and Encounter and Medicare Supplemental Databases. Area Health Resource Files provided non-clinical characteristics and sociodemographic data. Eligibility: Female sex, claim(s) with ICD-9-CM or ICD-10-CM diagnosis of non-metastatic invasive breast cancer, > 6 months of continuous insurance pre- and post-diagnosis, evidence of BCS or mastectomy following initial ICD9/10 code diagnosis. Logistic and quantile multivariable regression models assessed the association between clinical and non-clinical factors and the outcome of BCS and time to surgery, respectively.

**Results:**

A total of 53,060 women were included in the study. Compared to mastectomy, BCS was significantly associated with older age (ORs: 1.54 to 2.99, 95% CIs 1.45 to 3.38; ps < .0001) and higher community density of medical genetics (OR: 5.88, 95% CIs 1.38 to 25.00; *p* = 0.02) or obstetrics and gynecology (OR: 1.13, 95% CI 1.02 to 1.25; *p* = .02) physicians. Shorter time-to-BCS was associated with living in the South (−2.96, 95% CI −4.39 to −1.33; *p* < .0001). Longer time-to-BCS was associated with residence in more urban (4.18, 95% CI 0.08 to 8.29; *p* = 0. 05), educated (9.02, 95% CI 0.13 to 17.91; *p* = 0.05), or plastic-surgeon-dense (4.62, 95% CI 0.50 to 8.73; *p* = 0.03) communities.

**Conclusions:**

Clinical and non-clinical factors are associated with adoption of BCS and time to treatment, suggesting opportunities to ensure equitable and timely care.

**Supplementary Information:**

The online version contains supplementary material available at 10.1007/s10549-021-06155-1.

## Introduction

Over the last four decades, advances in detection, diagnosis and surgical treatment for non-metastatic breast cancer have resulted in improved survival and health outcomes [1–4]. Among these advances, is the acceptance of the survival equivalence of breast conserving surgery (BCS) with mastectomy [5–7]. Nevertheless, non-clinical factors such as health insurance status, sociodemographic characteristics, and availability of health care services are thought to influence use of BCS [8]. Likewise, multiple clinical factors affect time to surgical treatment including preoperative evaluations and the management of comorbidities [9].

While breast cancer guidelines recommend optimal time from diagnosis to initiation of radiation and chemotherapy, there is no such standard for time to surgery (TTS) [10]. A 2012 SEER-Medicare study of 72,586 women with invasive breast cancer documented mean and median times from presentation to surgery of 46 days and 29 days, respectively [11]. Follow-up studies in 2016 and 2019 by the same authors observed that survival outcomes for patients with early-stage breast cancer were impacted by a longer time between diagnosis and surgery [12, 13]. These previous studies have predominantly examined TTS from Medicare regional cancer registry populations, limiting their generalizability to commercially insured individuals. Significant differences in sociodemographic and clinical variables and outcomes, based on the type of insurance at presentation were identified by Obeng-Gyasi et al. [14].

Both the use of BCS and TTS have been described as indicative of care quality [13] in that quality of care includes obtaining appropriate, effective, and timely care [15]. Though evidence suggests clinical and non-clinical factors influence the use of BCS, the impact of non-clinical factors on TTS and on adoption of BCS versus mastectomy in contemporary evidence remains unclear [8, 16]. Thus, we hypothesized that clinical and non-clinical factors would be associated with the adoption of BCS and TTS. Specifically, our primary study objective aimed to examine whether and which clinical and non-clinical factors were associated with adoption of BCS and TTS in a large and diverse commercially insured sample of patients with non-metastatic invasive breast cancer. This paper describes: (1) national and regional trends in receipt of BCS versus mastectomy, (2) patient and community factors associated with receipt of BCS over mastectomy, (3) national and regional trends in time to primary surgical treatment, and (4) patient and community characteristics associated with time to primary surgical treatment.

## Methods

### Study design and data sources

This cross-sectional, retrospective, observational study utilized the MarketScan^®^ Commercial and Medicare Supplemental Databases to identify eligible patients. The MarketScan Commercial Database contains data on enrollment, select demographics, and the medical (inpatient and outpatient) and prescription-drug (outpatient only) claims of several million employees and their dependents covered under a variety of fee-for-service and capitated health plans. The MarketScan Medicare Supplemental Database contains the same health care experience data for individuals with Medicare supplemental insurance paid for by employers. All study data were obtained using International Classification of Diseases, 9th and 10th Revision, Clinical Modification (ICD-9 and ICD-10) codes, Current Procedural Terminology 4th edition (CPT) codes, Healthcare Common Procedure Coding System (HCPCS) codes, and National Drug Codes (NDCs). All database records were statistically de-identified and certified to be fully compliant with US patient confidentiality requirements set forth in the Health Insurance Portability and Accountability Act of 1996. As this study did not involve the collection, use, or transmittal of individually identifiable data, Institutional Review Board approval to conduct this study was not necessary.

The Area Health Resource Files (AHRF) were used to capture community-level characteristics on the availability of health care providers or services, income- and education-levels and sociodemographic composition of racial/ethnic groups. The AHRF is a publicly available dataset that aggregates information from more than 50 sources including the American Hospital Association Survey Database and the American Medical Association Physician Master File [17]. The 2019 AHRF data were linked to MarketScan via a crosswalk between U.S. county Federal Information Processing code and the policyholder’s ZIP3.

### Study sample selection

Female patients with one inpatient or two outpatient (non-diagnostic) medical claims of non-metastatic invasive breast cancer (ICD-9 code 174.x, ICD-10 codes C50011 or C50919) from January 1, 2012, to October 31, 2017 time period were considered for inclusion in the study. Patients ≥ 18 years of age on the date of the first observed medical claim for non-metastatic invasive breast cancer (the “index date”), continuously enrolled in the plan for at least 6 months pre- and post-index and having undergone breast conserving surgery or mastectomy within 6 months post-index were included in the sample. Patients with ductal carcinoma *in situ* (DCIS) diagnosis were included because this diagnosis is not uncommon prior to or at the time of invasive, non-metastatic breast cancer diagnosis. Furthermore, these individuals represent an important proportion of non-metastatic invasive breast cancer patients and the presence of co-existing DCIS may have a prognostic value in this patient population [18].

Patients were excluded if they had another cancer during the pre-index period, presented evidence of metastatic disease in the 6-month pre-index period through 90 days post-index, filled one or more prescriptions for medication primarily indicated for metastatic breast cancer treatment (e.g., abemaciclib, everolimus, olaparib), had a claim for any type of neoadjuvant therapy (chemotherapy, hormonal therapy, biologic therapy, or radiotherapy) or had missing data for demographic characteristics.

Medical and prescription claim codes were reviewed and confirmed by an expert panel of three practicing physicians (two internists and one oncologist). The expert panel reviewed descriptions of relevant claim code sets identified by a nosologist to approximate the following clinical evidence: non-metastatic invasive breast cancer diagnoses (ICD-9, ICD-10), surgical interventions of BCS and mastectomy (ICD-9, ICD-10, CPT), neoadjuvant therapies (ICD-9, ICD-10, HCPCS, NDC), and metastatic prescription drugs (NDC/HCPCS).

### Patient-level independent variables

The following clinical and non-clinical variables were constructed for each individual as of their index date for non-metastatic invasive breast cancer. Age (in years) was categorized as younger than 50, 50–59, 60–69, 70–79, and ≥ 80. Health plan type was grouped as: (1) preferred provider organization (PPO), point of service (POS) or comprehensive; (2) health maintenance organization (HMO) or exclusive provider organization (EPO); (3) consumer-directed health plans (CDHP); and (4) high-deductible health plans (HDHP). Region of patient residence was grouped as: Northeast, Midwest, South, and West. Binary variables were created for policyholder (versus spouse or other dependent) and data source contributor (employer versus health plan). Flags for year of diagnosis were also created (2012–2017).

To control for clinical presentation and baseline health status, several patient-specific measures (covariates) were derived including indicators of having had (1) BRCA 1/2 germline testing (genetic testing), (2) ductal carcinoma *in situ* (DCIS) diagnosis prior to the non-metastatic invasive breast cancer index date, or (3) concurrent DCIS diagnosis on the non-metastatic invasive breast cancer index date. Additionally, patients’ use of adjuvant therapies (chemotherapy, biologic therapy, hormonal therapy, and radiotherapy) within 6 months of surgical intervention were included as dichotomous variables. Finally, a vector of 15 indicators for the chronic health conditions captured by the Deyo-Charlson Comorbidity Index [19, 20] were also derived using medical claims data from the 6 months preceding the index date.

Non-clinical factors were represented by the patient-level variables of health plan, region of residence, data source contributor, policyholder status, and diagnosis year. The patient-level variables of age, genetic test, prior DCIS, index DCIS, use of adjuvant therapies, and Deyo-Charlson Comorbidity Index represented clinical factors that may influence BCS use and TTS.

### Community-level independent variables

Patient-level data were linked to county-level information based on the policyholder’s ZIP3. An expert panel of two physicians, an economist, and three health services researchers selected 15 community-specific sociodemographic and health care supply measures for inclusion in the analyses. Rurality was represented by the percentage of residents living in urban areas. Race/ethnicity was categorized into five groups: percentage Black, Asian, Hispanic, Other/Multi-Racial, and White. Educational attainment was specified as the percentage with a 4-year college degree, and income as median household income. Measures of health care availability in the community included physicians per 10,000 residents in five specialties: (1) obstetrics and gynecology, (2) plastic surgery, (3) medical genetics, (4) nuclear medicine, and (5) radiation oncology. Finally, the number of hospitals with general medicine surgical centers, as well as the number of hospitals with chemotherapy services (per 10,000 residents) were also captured. All community-level variables represented non-clinical factors hypothesized to influence the use of BCS and TTS.

### Dependent variables

Key outcomes of interest were the selection of BCS versus mastectomy (a dichotomous variable) and the elapsed time to surgery (a continuous variable measured in days). Patients with at least one BCS claim, and the absence of a mastectomy claim, 6 months following the index date were classified BCS. All others were defined as mastectomy patients. TTS was calculated as the difference in days between the date of service for the surgical intervention and the index date.

### Statistical analysis

Differences in means for all person-specific variables across selected surgery type were assessed using the Kruskal–Wallis equality of populations’ test [21]. Importantly, for all community-level measures, significant differences were evaluated using a variant of Somers’ D rank test, which accounted for correlation of errors at the ZIP3 level [22]. Multivariable logistic regression models were specified to assess the associations between all patient- and community-level factors previously described and the selection of BCS versus mastectomy. Reference categories (excluded from the model) were year = 2012, age < 50, Northeast region, plan type = PPO/POS/comprehensive, and percent white. Results were expressed as odds ratios (OR) for ease of interpretation. Again, p-values for the community-level independent variables were based on standard errors that were clustered by ZIP3 (number of clusters = 859). Marginal effects, the estimated impact on the likelihood of BCS, were also computed for all regressors at their mean values.

Since the TTS variables for BCS and mastectomy were not normally distributed, quantile regressions of the median were estimated as a function of all independent variables. Again, p-values for the community-level measures were based on standard errors that were clustered by ZIP3. A threshold of p < 0.05 was applied to identify statistical significance across analyses, and all statistical analyses were conducted using STATA/MP statistical software (version 16.0, StataCorp).

## Results

### Descriptive statistics

**Fig. 1:**
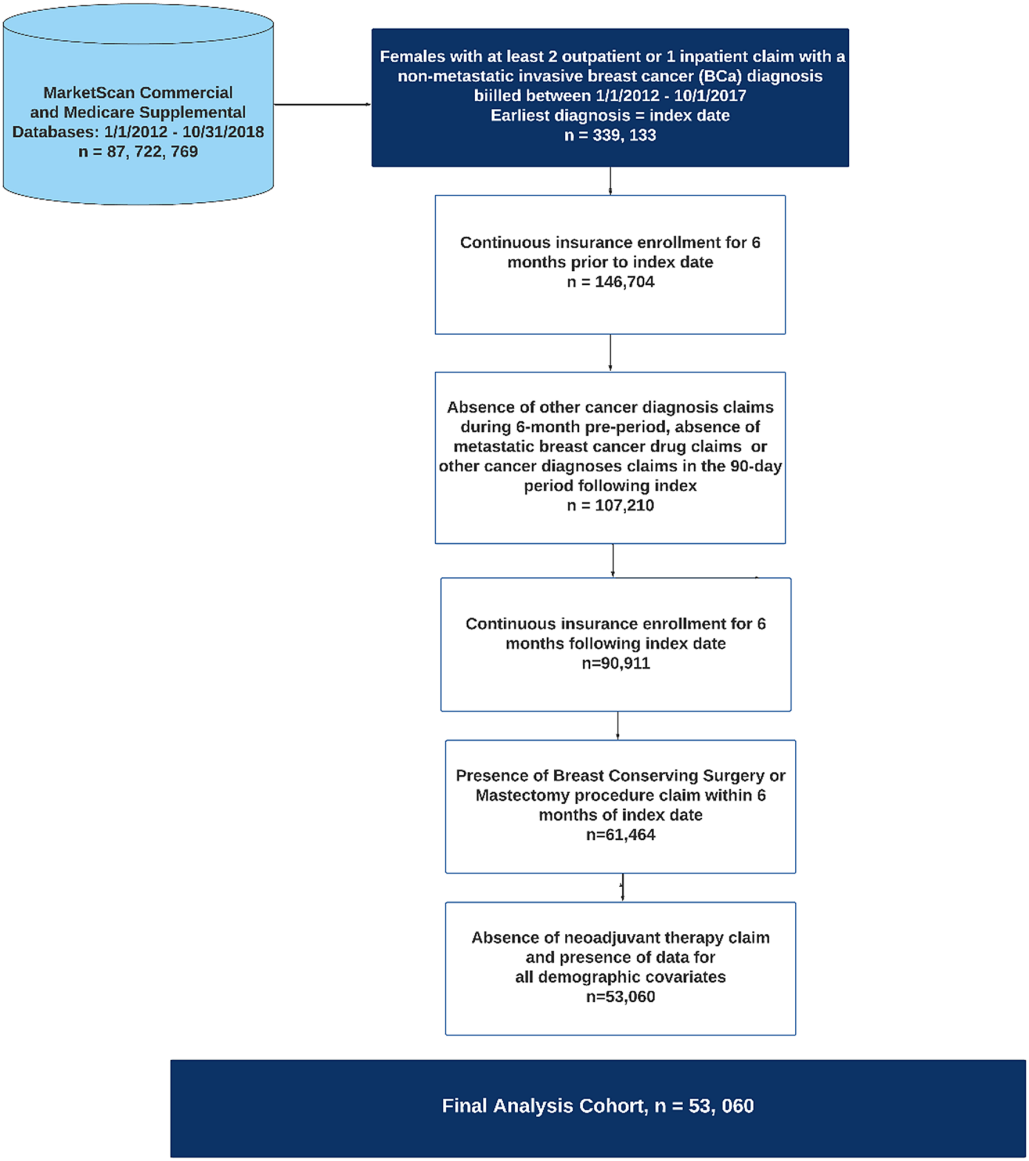
Sample selection

A total of 53,060 women were identified with a diagnosis of non-metastatic invasive breast cancer between January 1, 2012, and March 31, 2017 who had either a BCS or a mastectomy within six months following their initial diagnosis and met inclusion criteria (Fig. 1). Of these patients, 68.4% (*n* = 36,270) had BCS and 31.6% (*n* = 16,790) had a mastectomy. Over time, the proportion of patients who had BCS relative to mastectomies increased from 64.0% in 2012 to 74.5% in 2017 (*p* < 0.0001) (eFig. 1a). Further, across all U.S. geographical regions the proportion of patients who had BCS increased from 2012 to 2017. The Northeast consistently had the highest proportion of patients who had BCS (67.8% to 82.6%) and the South had the lowest (59.9% to 70.4%) (eTable 1).

Means and standard deviations for all variables by surgical intervention and overall are presented in Table 1. The majority of the overall cohort was 50 or older (77.6%), the policyholder (63.7%), enrolled in a PPO/POS/comprehensive health plan (74.1%), and lacked a genetic test (83.3%). Compared to mastectomy, BCS patients were on average older (59.7 vs. 55.4; *p* < 0.0001), and fewer had a prior DCIS diagnosis (25.3% vs. 30.6%; *p* < 0.001), adjuvant chemotherapy (14.2% vs. 18.5%; *p* < 0.001), and adjuvant hormonal therapy (25.6% vs. 33.2%; *p* < 0.001). Conversely, more BCS patients had adjuvant radiotherapy (49.6% vs. 2.0%; *p* < 0.001). Only one coexisting chronic condition was statistically significant: more BCS patients had diabetes mellitus compared to mastectomy patients (11.2% vs. 9.0%; *p* < 0.0001).

**Table 1:** Variable Means by breast cancer surgery type and overall cohort (*N* = 53,060)^a,b^

Variable	Overall (*N* = 53,060)		BCS (*N* = 36,270)		Mastectomy (*N* = 16,790)		*p*-value
	Mean	(sd)	Mean	(sd)	Mean	(sd)	
Year 2012	13.3%	(0.34)	12.4%	(0.33)	15.1%	(0.36)	0.00
Year 2013	24.2%	(0.43)	23.2%	(0.42)	26.6%	(0.44)	0.00
Year 2014	20.4%	(0.40)	19.8%	(0.40)	21.6%	(0.41)	0.00
Year 2015	17.6%	(0.38)	18.0%	(0.38)	16.7%	(0.37)	0.02
Year 2016	14.7%	(0.35)	15.9%	(0.37)	12.1%	(0.33)	0.00
Year 2017	9.8%	(0.30)	10.7%	(0.31)	7.9%	(0.27)	0.00
Age, years	58.4	(11.51)	59.7	(11.03)	55.4	(11.98)	0.00
Age < 50	22.4%	(0.42)	17.4%	(0.38)	33.1%	(0.47)	0.00
Age 50–59	33.6%	(0.47)	33.9%	(0.47)	33.0%	(0.47)	0.11
Age 60–69	27.6%	(0.45)	30.4%	(0.46)	21.6%	(0.41)	0.00
Age 70–79	11.3%	(0.32)	12.8%	(0.33)	8.1%	(0.27)	0.00
Age 80 +	5.1%	(0.22)	5.5%	(0.23)	4.2%	(0.20)	0.02
Employer-sponsored (versus Health plan)	79.8%	(0.40)	80.4%	(0.40)	78.6%	(0.41)	0.00
Policyholder	63.7%	(0.48)	64.4%	(0.48)	62.3%	(0.48)	0.00
Spouse	36.1%	(0.48)	35.5%	(0.48)	37.5%	(0.48)	0.00
Other dependent	0.1%	(0.03)	0.1%	(0.03)	0.2%	(0.04)	0.88
Northeast	20.0%	(0.40)	21.4%	(0.41)	17.0%	(0.38)	0.00
Midwest	24.3%	(0.43)	25.2%	(0.43)	22.3%	(0.42)	0.00
South	39.9%	(0.49)	37.5%	(0.48)	45.1%	(0.50)	0.00
West	15.8%	(0.36)	15.9%	(0.37)	15.6%	(0.36)	0.55
Plan Type: PPO, POS, or comprehensive	74.1%	(0.44)	74.4%	(0.44)	73.3%	(0.44)	0.03
Plan Type: EPO or HMO	12.2%	(0.33)	12.4%	(0.33)	11.7%	(0.32)	0.18
Plan Type: CDHP	7.9%	(0.27)	7.6%	(0.26)	8.6%	(0.28)	0.06
Plan Type: HDHP	4.6%	(0.21)	4.4%	(0.20)	5.1%	(0.22)	0.17
Had a Genetic Test	16.7%	(0.37)	13.1%	(0.34)	24.4%	(0.43)	0.00
Also Had In Situ on Index Date	18.9%	(0.39)	18.9%	(0.39)	19.1%	(0.39)	0.60
Also Had In Situ pre-Index Date	27.0%	(0.44)	25.3%	(0.43)	30.6%	(0.46)	0.00
Had cytotoxic chemotherapy post-surgery	15.6%	(0.36)	14.2%	(0.35)	18.5%	(0.39)	0.00
Had biologic therapy post-surgery	4.1%	(0.20)	3.8%	(0.19)	4.6%	(0.21)	0.13
Had hormonal therapy post-surgery	28.0%	(0.45)	25.6%	(0.44)	33.2%	(0.47)	0.00
Had radiation post-surgery	34.5%	(0.48)	49.6%	(0.50)	2.0%	(0.14)	0.00
*Charlson comorbidity index indicators*							
Congestive heart failure	1.1%	(0.10)	1.1%	(0.10)	1.0%	(0.10)	0.88
Chronic obstructive pulmonary disease	7.5%	(0.26)	7.8%	(0.27)	6.9%	(0.25)	0.07
Cerebrovascular disease	2.2%	(0.15)	2.4%	(0.15)	1.9%	(0.14)	0.38
Dementia	0.2%	(0.04)	0.2%	(0.04)	0.2%	(0.04)	0.95
Diabetes	10.5%	(0.31)	11.2%	(0.32)	9.0%	(0.29)	0.00
Diabetes + Complications	2.0%	(0.14)	2.3%	(0.15)	1.6%	(0.13)	0.22
AIDS	0.0%	(0.02)	0.0%	(0.02)	0.1%	(0.03)	0.95
Hemiplegia or Paraplegia	0.1%	(0.03)	0.1%	(0.03)	0.0%	(0.02)	0.88
Mild liver disease	0.2%	(0.04)	0.2%	(0.04)	0.2%	(0.04)	0.95
Moderate/severe liver disease	0.1%	(0.02)	0.1%	(0.02)	0.1%	(0.03)	0.95
Acute myocardial infarction	0.4%	(0.07)	0.4%	(0.07)	0.4%	(0.06)	0.88
Peptic ulcer	0.3%	(0.05)	0.3%	(0.06)	0.3%	(0.05)	0.95
Peripheral vascular disease	1.1%	(0.11)	1.3%	(0.11)	0.9%	(0.09)	0.47
Renal disease	1.8%	(0.13)	2.0%	(0.14)	1.4%	(0.12)	0.24
Rheumatoid disease	1.5%	(0.12)	1.5%	(0.12)	1.4%	(0.12)	0.84
*ZIP3-level variables*							
Percent Urban	89.8%	(0.14)	90.0%	(0.14)	89.5%	(0.14)	0.31
Percent White	59.1%	(0.19)	59.4%	(0.19)	58.5%	(0.19)	0.01
Percent Black	17.0%	(0.13)	16.8%	(0.13)	17.5%	(0.13)	0.05
Percent Asian	6.2%	(0.07)	6.3%	(0.07)	6.1%	(0.06)	0.01
Percent Hispanic	14.1%	(0.14)	13.9%	(0.13)	14.5%	(0.14)	0.38
Percent other race/ethnicity	3.5%	(0.02)	3.5%	(0.02)	3.5%	(0.02)	0.26
Percent with 4-year college degree	34.7%	(0.10)	34.8%	(0.10)	34.4%	(0.10)	0.09
Median household income ($10 thousands)	$5.86	(1.51)	$5.90	(1.52)	$5.79	(1.48)	0.01
# Ob-gyn physicians (per 10 k residents)	156.5%	(0.68)	156.4%	(0.68)	156.7%	(0.68)	0.78
# Plastic surgery physicians (per 10 k residents)	33.8%	(0.22)	33.4%	(0.22)	34.5%	(0.22)	0.00
# Diagnostic radiology physicians (per 10 k residents)	106.6%	(0.59)	106.6%	(0.59)	106.6%	(0.59)	0.72
# Medical genetics physicians (per 10 k residents)	2.7%	(0.03)	2.7%	(0.03)	2.6%	(0.03)	0.03
# Nuclear medicine physicians (per 10 k residents)	4.5%	(0.05)	4.6%	(0.05)	4.4%	(0.05)	0.00
# Radiation oncology physicians (per 10 k residents)	21.4%	(0.14)	21.4%	(0.14)	21.5%	(0.14)	0.15
# Hospitals with general medicine/surgical center (per 10 k residents)	7.7%	(0.06)	7.7%	(0.05)	7.9%	(0.06)	0.03
# Hospitals with chemotherapy (per 10 k residents)	6.3%	(0.05)	6.2%	(0.05)	6.4%	(0.05)	0.35

^a^p-values are from Kruskal–Wallis equality of populations tests across surgery type (clustered by ZIP3 using Somers' D rank test where appropriate)

^b^All values are proportions unless denoted otherwise

*BCS* breast conserving surgery, *SD* standard deviation, *PPO* preferred provider organization, *POS* point of service, *EPO* exclusive provider organization, *HMO* health maintenance organization, *CDHP* consumer driven health insurance, *HDHP* high-deductible health plan, *AIDS* acquired immune deficiency syndrome, *OB-GYN* obstetrics and gynecology

Table 2 reports results from the logistic model of BCS compared to mastectomy. Associations were identified between the majority of patient- and community-level factors and BCS. Compared to mastectomy patients, BCS patients were more likely to have been in an age group older than 50 (ORs: 1.54 to 2.99, 95% CIs 1.45 to 3.38; ps < 0.001), receive adjuvant radiotherapy (OR: 61.30, 95% CI 54.27 to 69.23; *p* < 0.001) or receive adjuvant chemotherapy (OR: 1.45, 95% CI 1.36 to 1.55; *p* < 0.001). The odds of receiving BCS over mastectomy were also significantly higher for patients living in communities with greater numbers of physicians (per 10,000 residents) in the specialties of medical genetics (OR: 5.88, 95% CI 1.38 to 25.00; *p* = 0.02) or obstetrics and gynecology (OR: 1.13, 95% CI 1.02 to 1.25; *p* = 0.02). Also, communities with a higher median household income (per $10,000) had an increased likelihood of BCS (OR: 1.04, 95% CI 1.00 to 1.09; *p* = 0.04).

**Table 2:** Logistic regression model of receiving breast conserving surgery versus mastectomy (*N* = 53,060)^a,b^

Variable	Odds ratio	*p*-value	[95% Confidence interval]
Year 2013	1.07	0.074	*[0.99, 1.15]*
Year 2014	1.14	0.001	*[1.05, 1.22]*
Year 2015	1.43	0.000	*[1.32, 1.55]*
Year 2016	1.78	0.000	*[1.63, 1.96]*
Year 2017	1.93	0.000	*[1.74, 2.13]*
Age 50–59	1.54	0.000	*[1.45, 1.64]*
Age 60–69	2.00	0.000	*[1.87, 2.14]*
Age 70–79	2.56	0.000	*[2.33, 2.82]*
Age 80 +	2.99	0.000	*[2.65, 3.38]*
Employer-sponsored (versus health plan)	1.03	0.458	*[0.95, 1.11]*
Policyholder (versus spouse or other dependent)	1.06	0.022	*[1.01, 1.11]*
Midwest region	0.82	0.000	*[0.74, 0.90]*
South region	0.63	0.000	*[0.56, 0.70]*
West region	0.77	0.000	*[0.68, 0.86]*
Plan type: EPO or HMO	1.11	0.017	*[1.02, 1.20]*
Plan type: CDHP	1.00	0.923	*[0.92, 1.08]*
Plan type: HDHP	0.90	0.046	*[0.82, 1.00]*
Had a genetic test	0.62	0.000	*[0.59, 0.67]*
Also Had In Situ on index date	1.03	0.286	*[0.97, 1.10]*
Also Had In Situ pre-index date	0.73	0.000	*[0.69, 0.76]*
Had cytotoxic chemotherapy post-surgery	1.45	0.000	*[1.36, 1.55]*
Had biologic therapy post-surgery	1.00	0.982	*[0.90, 1.11]*
Had hormonal therapy post-surgery	0.46	0.000	*[0.43, 0.49]*
Had radiation post-surgery	61.30	0.000	*[54.27, 69.23]*
*Charlson comorbidity index indicators*			
Congestive heart failure	0.81	0.047	*[0.65, 1.00]*
Chronic obstructive pulmonary disease	1.07	0.071	*[0.99, 1.16]*
Cerebrovascular disease	1.00	0.973	*[0.86, 1.15]*
Dementia	0.56	0.039	*[0.33, 0.97]*
Diabetes	1.06	0.165	*[0.98, 1.15]*
Diabetes + Complications	1.16	0.083	*[0.98, 1.38]*
AIDS	0.84	0.673	*[0.38, 1.87]*
Hemiplegia or paraplegia	1.90	0.188	*[0.73, 4.91]*
Mild liver disease	0.81	0.447	*[0.47, 1.40]*
Moderate/severe liver disease	0.72	0.052	*[0.26, 1.96]*
Acute myocardial infarction	1.19	0.300	*[0.86, 1.66]*
Peptic ulcer	0.87	0.523	*[0.57, 1.33]*
Peripheral vascular disease	1.09	0.435	*[0.88, 1.33]*
Renal disease	1.19	0.057	*[1.00, 1.41]*
Rheumatoid disease	1.01	0.941	*[0.84, 1.21]*
*ZIP3-level variables*			
Percent Urban	0.92	0.555	*[0.69, 1.22]*
Percent Black	1.12	0.634	*[0.71, 1.76]*
Percent Asian	1.35	0.415	*[0.66, 2.76]*
Percent Hispanic	1.04	0.843	*[0.72, 1.48]*
Percent other race/ethnicity	3.13	0.099	*[0.81, 12.14]*
Percent with 4-year college degree	0.91	0.804	*[0.44, 1.90]*
Median household income ($10 thousands)	1.04	0.044	*[1.00, 1.09]*
# Ob-gyn physicians (per 10 k residents)	1.13	0.022	*[1.02, 1.25]*
# Plastic surgery physicians (per 10 k residents)	0.69	0.007	*[0.52, 0.90]*
# Diagnostic radiology physicians (per 10 k residents)	0.91	0.132	*[0.80, 1.03]*
# Medical genetics physicians (per 10 k residents)	5.88	0.016	*[1.38, 25.00]*
# Nuclear medicine physicians (per 10 k residents)	2.43	0.071	*[0.93, 6.35]*
# Radiation oncology physicians (per 10 k residents)	0.89	0.634	*[0.54, 1.45]*
# Hospitals with general medicine/surgical center (per 10 k residents)	0.28	0.010	*[0.11, 0.74]*
# Hospitals with chemotherapy (per 10 k residents)	1.76	0.303	*[0.60, 5.20]*
Constant	0.73	0.079	*[0.51, 1.04]*

^a^p-values for ZIP3-level variables are based on clustered standard errors

^b^Reference categories include: Year = 2012, Age < 50, Northeast Region, PPO/POS/Comprehensive Health Plan Type, Percent White

*EPO* exclusive provider organization, *HMO* health maintenance organization, *CDHP* consumer driven health insurance, *HDHP* high-deductible health plan, *AIDS* acquired immune deficiency syndrome, *Ob-GYN* obstetrics and gynecology

Factors associated with reduced odds of BCS included having adjuvant hormonal therapy (OR: 0.46, 95% CI 0.43 to 0.49; *p* < 0.0001) or a genetic test (OR: 0.62, 95% CI 0.59 to 0.67); *p* < 0.001); residing in the South, Midwest, or West (OR: 0.63 to 0.82, 95% CI 0.56 to 0.90; ps < 0.001); and a greater availability of plastic surgeons (OR: 0.69, 95% CI 0.52 to 0.90; *p* = 0.01) or hospitals with general medicine/surgical centers (OR: 0.28, 95% CI 0.11 to 0.74; p = 0.01) (eFig. 2). Interestingly, patients with dementia were 43.7% less likely (OR: 0.56, 95% CI 0.33 to 0.97; *p* = 0.04) to have received BCS compared to mastectomy.

### Time to surgery

For BCS, the median [Q1, Q3] TTS (in days) increased from 21.0 [11, 33] in 2012 to 25 [14, 36] in 2017 (eFig. 1b, eTable 2). From the multivariable quantile regression model (Table 3), BCS patients had shorter TTS if they were living in the South (−2.86, 95% CI −4.39 to −1.33; *p* < 0.001), had a prior DCIS diagnosis (−8.96, 95% CI −9.58 to −8.35; *p* < 0.001), had a concurrent DCIS diagnosis (−4.84, 95% CI −5.60 to −4.09; *p* < 0.001), or had adjuvant chemotherapy (−1.90, 95% CI −2.59 to −1.22; *p* < 0.001). Longer BCS TTS was experienced by those living in more educated (9.02, 95% CI 0.13 to 17.91; *p* = 0.05), urban (4.18, 95% CI 0.08 to 8.29; *p* = 0.05) or plastic-surgeon-dense communities (4.62, 95% CI 0.50 to 8.73; *p* = 0.03); having a genetic test (7.41, 95% CI 6.68 to 8.15; *p* < 0.001), adjuvant hormonal therapy (0.93, 95% CI 0.42 to 1.44; *p* < 0.01) or adjuvant biologic therapy (1.40, 95% CI 0.34 to 2.45; *p* = 0.01); having any of the following comorbid conditions: congestive heart failure (2.24, 95% CI 0.68 to 3.81; p = 0.01), cerebrovascular disease (1.93, 95% CI 0.66 to 3.21; p < 0.01), renal disease 2.10, 95% CI 0.58 to 3.63; p = 0.01), chronic obstructive pulmonary disease (0.99, 95% CI 0.33 to 1.65; p < 0.01), or dementia (4.29, 95% CI: 0.02 to 8.57; *p* = 0.05).

**Table 3:** Quantile (Median) regression of days to breast conserving surgery (*N* = 36,270)^a,b^

Variable	Coefficient	[95% Confidence interval]		*p*-value
Year 2013	0.60	−*[0.08, 1.29]*		0.09
Year 2014	2.11	*[1.35, 2.87]*		0.00
Year 2015	2.80	*[2.00, 3.61]*		0.00
Year 2016	3.02	*[2.10, 3.93]*		0.00
Year 2017	4.11	*[3.20, 5.01]*		0.00
Age 50–59	−0.23	−*[0.95, 0.48]*		0.53
Age 60–69	0.00	−*[0.67, 0.67]*		0.99
Age 70–79	0.08	−*[0.79, 0.95]*		0.86
Age 80 +	−0.59	−*[1.80, 0.62]*		0.34
Employer-sponsored (versus Health Plan)	0.47	−*[0.39, 1.33]*		0.29
Policyholder (versus spouse or other dependent)	0.15	−*[0.34, 0.64]*		0.55
Midwest region	−0.57	−*[2.25, 1.11]*		0.51
South region	−2.86	−*[4.39, *−*1.33]*		0.00
West region	−1.33	−*[3.26, 0.59]*		0.17
Plan type: EPO or HMO	0.16	−*[1.01, 1.33]*		0.79
Plan type: CDHP	0.23	−*[0.63, 1.08]*		0.60
Plan type: HDHP	−0.02	−*[1.17, 1.13]*		0.97
Had a genetic test	7.41	*[6.68, 8.15]*		0.00
Also had In Situ on index date	−4.84	−*[5.60* −*, 4.09]*		0.00
Also had In Situ pre-index date	−8.96	−*[9.58, *−*8.35]*		0.00
Had cytotoxic chemotherapy post-surgery	−1.90	−*[2.59, *−*1.22]*		0.00
Had biologic therapy post-surgery	1.40	*[0.34, 2.45]*		0.01
Had hormonal therapy post-surgery	0.93	*[0.42, 1.44]*		0.00
Had radiation post-surgery	0.07	−*[0.46, 0.60]*		0.80
*Charlson comorbidity index indicators*				
Congestive heart failure	2.24	*[0.68, 3.81]*		0.01
Chronic obstructive pulmonary disease	0.99	*[0.33, 1.65]*		0.00
Cerebrovascular disease	1.93	*[0.66, 3.21]*		0.00
Dementia	4.29	*[0.02, 8.57]*		0.05
Diabetes	0.72	−*[0.17, 1.62]*		0.11
Diabetes + Complications	−0.02	−*[1.37, 1.33]*		0.98
AIDS	3.41	−*[2.19, 9.01]*		0.23
Hemiplegia or Paraplegia	1.60	−*[4.96, 8.16]*		0.63
Mild Liver disease	−0.98	−*[3.87, 1.92]*		0.51
Moderate/severe liver disease	0.39	−*[6.70, 7.47]*		0.92
Acute myocardial infarction	0.56	−*[3.08, 4.19]*		0.77
Peptic ulcer	−0.13	−*[2.23, 1.98]*		0.91
Peripheral vascular disease	0.61	−*[0.92, 2.14]*		0.44
Renal disease	2.10	*[0.58, 3.63]*		0.01
Rheumatoid disease	−0.73	−*[2.65, 1.19]*		0.46
*ZIP3-level variables*				
Percent Urban	4.18	*[0.08, 8.29]*		0.05
Percent Black	0.29	−*[5.80, 6.37]*		0.93
Percent Asian	0.17	−*[11.06, 11.40]*		0.98
Percent Hispanic	3.79	−*[1.14, 8.72]*		0.13
Percent other race/ethnicity	−2.99	−*[21.49, 15.50]*		0.75
Percent with 4-year college degree	9.02	*[0.13, 17.91]*		0.05
Median household income ($10 thousands)	−0.45	−*[1.14, 0.23]*		0.19
# Ob-gyn physicians (per 10 k residents)	−0.59	−*[1.79, 0.61]*		0.33
# Plastic surgery physicians (per 10 k residents)	4.62	*[0.50, 8.73]*		0.03
# Diagnostic radiology physicians (per 10 k residents)	−1.25	−*[2.85, 0.35]*		0.13
# Medical genetics physicians (per 10 k residents)	5.69	−*[13.34, 24.73]*		0.56
# Nuclear medicine physicians (per 10 k residents)	7.80	−*[9.68, 25.29]*		0.38
# Radiation oncology physicians (per 10 k residents)	−3.66	−*[10.46, 3.15]*		0.29
# Hospitals with general medicine/surgical center (per 10 k residents)	−5.31	−*[15.57, 4.94]*		0.31
# Hospitals with chemotherapy (per 10 k residents)	2.46	−*[8.81, 13.72]*		0.67
Constant	19.56	*[13.66, 25.46]*		0.00

^a^p-values for ZIP3-level variables are based on clustered standard errors

^b^Reference categories include: Year = 2012, Age < 50, Northeast Region, PPO/POS/Comprehensive Health Plan Type, Percent White

*EPO* exclusive provider organization, *HMO* health maintenance organization, *CDHP* consumer driven health insurance, *HDHP* high-deductible health plan, *AIDS* acquired immune deficiency syndrome, *OB-GYN* obstetrics and gynecology

For mastectomy (eFig. 1c, eTable 3), median TTS increased from 31.0 [19, 48] in 2012 to 35.5 [21, 51] in 2017. Shorter mastectomy TTS was related to being in an age group 60 and older (−8.80 to −1.88, 95% CI −11.06 to −0.72; *p* < 0.001 to *p* = 0.001); having a prior DCIS diagnosis (−6.66, 95% CI −7.59 to −5.73; *p* < 0.001); receiving adjuvant chemotherapy (−6.23, 95% CI −7.48 to −4.97; *p* < 0.001); receiving adjuvant hormonal therapy (−1.52, 95% CI −2.45 to −0.59; *p* = 0.001); residing in the Midwest (−2.96, 95% CI −5.13 to −0.79; *p* = 0.01), South (−4.69, 95% CI −6.69 to −2.70; *p* < 0.001), West (−6.17, 95% CI −8.59 to −3.75; *p* < 0.001), or in communities with a higher number of radiation oncologists (−12.12, −20.07 to −4.16; *p* < 0.01) (Table 4, eFigs. 3, 4).

**Table 4:** Quantile (Median) regression of days to mastectomy (*N* = 16,790)^a,b^

Variable	Coefficient	[95% Confidence interval]	*p*-value
Year 2013	0.29	−*[0.96, 1.54]*	0.65
Year 2014	1.37	*[0.00, 2.74]*	0.05
Year 2015	2.06	*[0.69, 3.42]*	0.00
Year 2016	2.26	*[0.59, 3.93]*	0.01
Year 2017	2.70	*[0.84, 4.57]*	0.01
Age 50–59	−0.27	−*[1.32, 0.77]*	0.61
Age 60–69	−1.88	−*[3.04, *−*0.72]*	0.00
Age 70–79	−6.03	−*[7.61* −*, 4.46]*	0.00
Age 80 +	−8.80	−*[11.06, *−*6.55]*	0.00
Employer-sponsored (versus health plan)	0.11	−*[1.11, 1.33]*	0.86
Policyholder (versus spouse or other dependent)	0.09	−*[0.74, 0.91]*	0.84
Midwest region	−2.96	−*[5.13, *−*0.79]*	0.01
South region	−4.69	−*[6.69, *−*2.70]*	0.00
West region	−6.17	−*[8.59, *−*3.75]*	0.00
Plan type: EPO or HMO	2.40	*[0.77, 4.03]*	0.00
Plan type: CDHP	1.43	*[0.04, 2.83]*	0.04
Plan type: HDHP	1.59	−*[0.24, 3.42]*	0.09
Had a genetic test	4.44	*[3.43, 5.44]*	0.00
Also had In Situ on index date	−0.25	−*[1.30, 0.81]*	0.65
Also had In Situ pre-index date	−6.66	−*[7.59, *−*5.73]*	0.00
Had cytotoxic chemotherapy post-surgery	−6.23	−*[7.48, *−*4.97]*	0.00
Had biologic therapy post-surgery	1.10	−*[0.76, 2.95]*	0.25
Had hormonal therapy post-surgery	−1.52	−*[2.45, *−*0.59]*	0.00
Had radiation post-surgery	−1.71	−*[5.52, 2.09]*	0.38
*Charlson comorbidity index indicators*			
Congestive heart failure	0.98	−*[2.61, 4.57]*	0.59
Chronic obstructive pulmonary disease	−0.33	−*[1.77, 1.11]*	0.65
Cerebrovascular disease	−1.27	−*[3.40, 0.87]*	0.25
Dementia	0.66	−*[4.07, 5.40]*	0.78
Diabetes	1.17	−*[0.26, 2.60]*	0.11
Diabetes + Complications	−0.91	−*[4.19, 2.38]*	0.59
AIDS	9.28	*[0.34, 18.21]*	0.04
Hemiplegia or paraplegia	4.20	−*[4.49, 12.89]*	0.34
Mild liver disease	−2.60	−*[9.19, 3.99]*	0.44
Moderate/severe liver disease	1.16	−*[10.41, 12.73]*	0.84
Acute myocardial infarction	−2.01	−*[7.17, 3.16]*	0.45
Peptic ulcer	1.29	−*[4.28, 6.85]*	0.65
Peripheral vascular disease	3.67	−*[2.02, 9.37]*	0.21
Renal disease	−0.62	−*[5.18, 3.94]*	0.79
Rheumatoid disease	1.22	−*[1.54, 3.98]*	0.39
*ZIP3-level variables*			
Percent Urban	12.78	*[7.88, 17.69]*	0.00
Percent Black	−5.97	−*[13.72, 1.79]*	0.13
Percent Asian	2.05	−*[10.81, 14.91]*	0.76
Percent Hispanic	1.83	−*[4.73, 8.38]*	0.59
Percent other race/ethnicity	−13.80	−*[34.71, 7.12]*	0.20
Percent with 4-year college degree	11.62	−*[2.51, 25.74]*	0.11
*Variable*		*[95% Confidence interval]*	*p-value*
Median household income ($10 thousands)	−0.72	−*[1.65, 0.21]*	0.13
# Ob-gyn physicians (per 10 k residents)	0.21	−*[1.91, 2.33]*	0.85
# Plastic surgery physicians (per 10 k residents)	3.19	−*[1.86, 8.23]*	0.22
# Diagnostic radiology physicians (per 10 k residents)	−0.57	−*[2.59, 1.45]*	0.58
# Medical genetics physicians (per 10 k residents)	22.08	−*[6.92, 51.08]*	0.14
# Nuclear medicine physicians (per 10 k residents)	−0.09	−*[19.14, 18.97]*	0.99
# Radiation oncology physicians (per 10 k residents)	−12.12	−*[20.07, *−*4.16]*	0.00
# Hospitals with general medicine/surgical center (per 10 k residents)	0.30	−*[15.92, 16.52]*	0.97
# Hospitals with chemotherapy (per 10 k residents)	−14.46	−*[32.35, 3.43]*	0.11
Constant	30.95	*[25.05, 36.84]*	0.00

^a^p-values for ZIP3-level variables are based on clustered standard errors

^b^Reference categories include: Year = 2012, Age < 50, Northeast Region, PPO/POS/Comprehensive Health Plan Type, Percent White

*EPO* exclusive provider organization, *HMO* health maintenance organization, *CDHP* consumer driven health insurance, *HDHP* high-deductible health plan, *AIDS* acquired immune deficiency syndrome, *OB-GYN* obstetrics and gynecology

Mastectomy patients in HMO/EPO plans had longer TTS (2.40, 95% CI 0.77 to 4.03; *p* < 0.01) as did those enrolled in CDHPs (1.43, 95% CI 0.04 to 2.83; *p* = 0.04). AIDS was one chronic condition significantly associated with a longer TTS (9.28, 95% CI 0.34 to 18.21; *p* = 0.04). Lastly, patients residing in communities that had a greater percent urban population had longer TTS for mastectomy (12.78, 95% CI 7.88 to 17.69; *p* < 0.001).

## Discussion

In this retrospective observational study of a large and diverse sample of commercially insured female patients with non-metastatic invasive breast cancer, we identified several clinical and non-clinical (i.e., sociodemographic) factors associated with the likelihood of receiving BCS. We also noted multiple factors that correlated with longer TTS across US geographic regions. Between 2012 and 2017, there was a 16% increase in the proportion of patients undergoing BCS relative to mastectomy. Strikingly, older patients were at least 54% more likely than younger patients to undergo BCS. Moreover, patients residing in areas with a higher density of medical geneticists and obstetricians and gynecologists had a significantly higher likelihood of undergoing BCS. The strong association between BCS and receipt of adjuvant radiotherapy was expected since it is a standard treatment after BCS.

The gradual increase in the proportion of patients undergoing BCS in our cohort aligns with increasing adherence to guideline-based surgical recommendations [23]. Since the 1991 National Institutes of Health consensus statement [24], level 1 evidence has demonstrated no difference in long-term survival between BCS followed by adjuvant radiotherapy to the ipsilateral breast and mastectomy in patients with non-metastatic invasive breast cancer [25, 26]. The finding that women ages 50 years and older were more likely to undergo BCS compared to patients under 50 years cannot be explained by our study but is most likely multifactorial. Some studies have shown that advancing age may influence the presentation and biologic behavior of breast cancer [27]. It is plausible that a higher proportion of such older patients with more favorable breast cancer features at presentation undergo BCS as compared to their younger counterparts. Younger, pre-menopausal patients may undergo mastectomy to lower their risk of ipsilateral breast cancer recurrence from somatic [28] or inherited genetic alterations such as BRCA1/2 germline mutations [29].

The reasons for the positive association between BCS and residence in areas with higher numbers of medical geneticists or gynecologists and obstetricians or higher median income cannot be derived from the current retrospective study. Nevertheless, it is plausible that these patients had access to more comprehensive multidisciplinary breast cancer services, including second opinion, and greater information about management options [10].

The increase in TTS we observed could be explained by an increase in preoperative evaluation workup, including MRI, genetic counseling, fertility counseling, and psychosocial assessment for distress [23], particularly for patients with higher educational status and with more urban residence where health services are more readily accessible. Future studies should further examine the non-clinical factors identified by this study as associated with BCS and/or TTS in a non-administrative dataset for a large commercial population.

Our study results suggest a nuanced approach to considering TTS as a stand-alone quality measure. Patient multidisciplinary evaluation and local–regional treatment planning enhance the quality of non-metastatic invasive breast cancer care [30] and patient satisfaction, however, the logistics of coordinating these services may be associated with unintended delays in scheduling surgery. Increasing utilization of multidisciplinary care, particularly in settings of reduced physician capacity, may contribute to TTS prolongation in some patients [31, 32]. Other potential factors include a possible growth in the number of second opinions and demand for surgical treatment for non-metastatic breast cancer without a parallel increase in services in this population. For example, females with a BRCA1/2 genetic test had longer TTS for BCS as well as mastectomy. While plausible, each of these explanations remains hypothetical and needs further evaluation in confirmatory studies. Further, considering a nuanced approach to TTS (i.e., shorter TTS may not equate to higher quality) suggests the opportunity for clinical practice standards that more explicitly define the timeliness of surgical intervention for non-metastatic invasive breast cancer to optimize patient outcomes.

### Limitations

Analysis data were derived from large national claims databases. Consequently, these results may not be generalizable to patients with Medicaid or no health insurance coverage. Also, these data are collected to facilitate payment for medical services and lack the clinical granularity found in medical records (e.g., lacking cancer staging, biologic subtype, family history); therefore, the potential for misclassification and/or coding errors is inherent. Multivariable modeling was limited to characteristics that can be measured from administrative claims. As a cross-sectional study, the results described reflect associations and do not imply causality. Also, various clinical or non-clinical characteristics may have affected the results of this study. For example, patient preference for BCS vs. mastectomy was not assessed. Patients with previously diagnosed/treated DCIS or concurrent breast reconstruction surgery, which may potentially alter their TTS, were not excluded from analyses. Receipt of adjuvant therapy was only captured 2 months post-surgery, potentially underestimating actual utilization.

## Conclusions

Health care service availability and multiple sociodemographic factors were consistently associated with preferential BCS use and TTS. While treatment planning in non-metastatic invasive breast cancer has become increasingly complex with greater use of imaging, second opinions, clinical decision-support tools, and multidisciplinary tumor boards, this study identified several non-clinical factors associated with BCS and TTS. Understanding the influence of non-clinical factors such as area of residence and patient sociodemographic characteristics on BCS and TTS are important considerations to ensure equitable access to breast cancer care and outcomes.

## Supplementary Information

Below is the link to the electronic supplementary material.

Supplementary file1 (DOCX 2051 KB)

## Data Availability

The Area Health Resource Files is a publicly available dataset. Marketscan is a proprietary database accessible via subscription.
