# Supplementary material for: Variations in breast cancer surgical treatment and timing: determinants and disparities
Source: Breast Cancer Res Treat. 2021 Mar 10;188(1):259–72. doi: 10.1007/s10549-021-06155-1 (PMC8233284; doi:10.1007/s10549-021-06155-1)
Supplement: Supplementary file 1 — Supplementary file1 (DOCX 2051 KB) [file 10549_2021_6155_MOESM1_ESM.docx]

**Variations in Breast Cancer Surgical Treatment and Timing: Determinants and Disparities Online Supplement**

eFigure 1. 2012–2017 Trends in Rates and Time to Breast Conserving Surgery versus Mastectomy by Geographical Region

eTable 1. Mean Rates of Breast Conserving Surgery versus Mastectomy

eFigure 2. Logistic Model Results of Receiving Breast Conserving Surgery versus Mastectomy

eTable 2. Trends in Median Time (in Days) to Breast Conserving Surgery

eTable 3. Trends in Median Time (in Days) to Mastectomy

eFigure 3. Quantile (Median) Regression of Days to Breast Conserving Surgery

eFigure 4. Quantile (Median) Regression of Days to Mastectomy

**eFigure 1. 2012–2017 Trends in Rates and Time to Breast Conserving Surgery versus Mastectomy by Geographical Region**


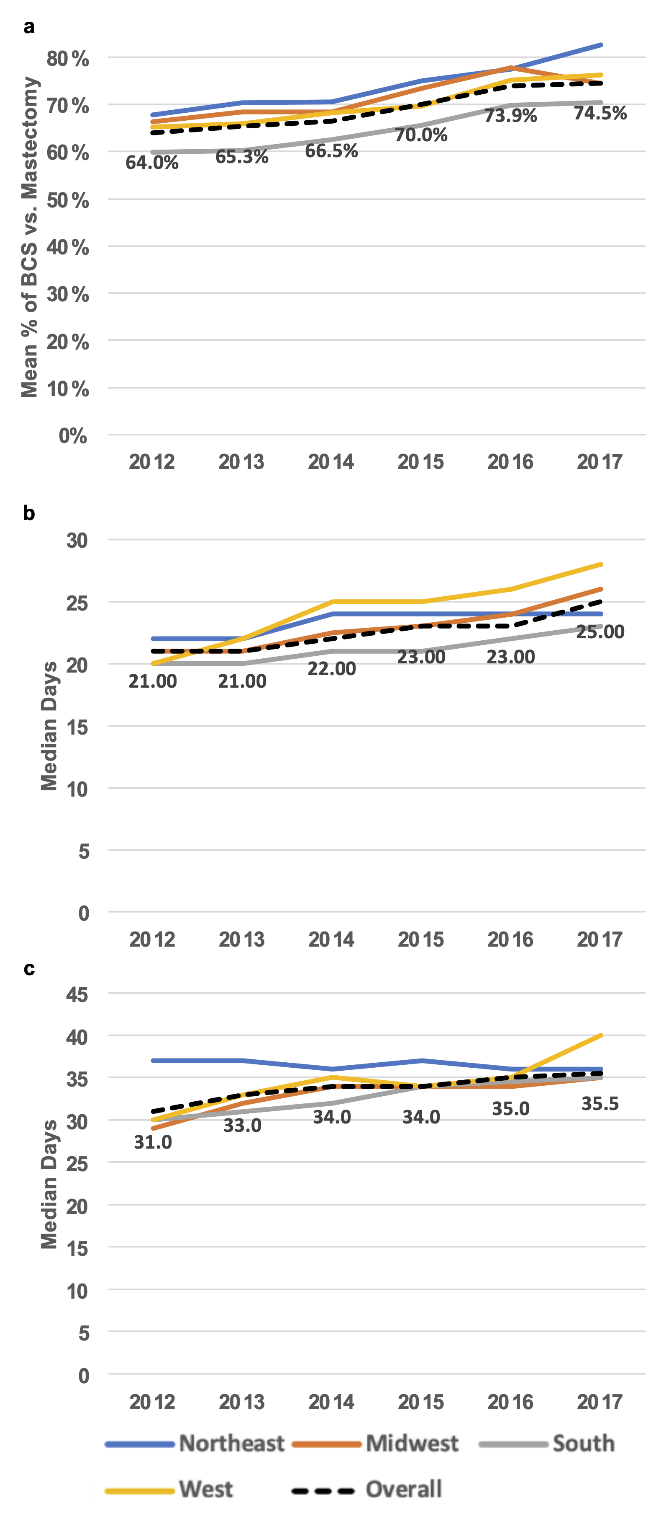


a) Mean rates of breast conserving surgery versus mastectomy (N=53,060), b) median days from first breast cancer diagnosis to breast conserving surgery (N=36,270), c) median days from first breast cancer diagnosis to mastectomy (N=16,790)

**eTable 1. Mean Rates of Breast Conserving Surgery versus Mastectomy^a^**

| **Region** | **2012** | | | **2013** | | | **2014** | | | **2015** | | | **2016** | | | **2017** | | | **p-value** |
| --- | --- | --- | --- | --- | --- | --- | --- | --- | --- | --- | --- | --- | --- | --- | --- | --- | --- | --- | --- |
|  | **n** | **mean** | **sd** | **n** | **mean** | **sd** | **n** | **mean** | **sd** | **n** | **mean** | **sd** | **n** | **mean** | **sd** | **n** | **mean** | **sd** |  |
| NE | 1,381 | 67.8% | 0.47 | 2,608 | 0.70 | 0.46 | 2,281 | 70.6% | 0.46 | 1,832 | 74.9% | 0.43 | 1,483 | 77.5% | 0.42 | 1,024 | 82.6% | 0.38 | <0.001 |
| MW | 1,624 | 66.3% | 0.47 | 3,159 | 0.68 | 0.46 | 2,637 | 68.4% | 0.46 | 2,407 | 73.3% | 0.44 | 1,895 | 77.8% | 0.42 | 1,178 | 74.4% | 0.44 | <0.001 |
| South | 2,628 | 59.9% | 0.49 | 4,753 | 0.60 | 0.49 | 4,316 | 62.5% | 0.48 | 3,813 | 65.5% | 0.48 | 3,373 | 69.8% | 0.46 | 2,284 | 70.4% | 0.46 | <0.001 |
| West | 1,425 | 65.2% | 0.48 | 2,343 | 0.66 | 0.47 | 1,574 | 68.2% | 0.47 | 1,281 | 69.6% | 0.46 | 1,062 | 75.1% | 0.43 | 699 | 76.3% | 0.43 | <0.001 |
| **Overall** | **7,058** | **64.0%** | **0.48** | **12,863** | **0.65** | **0.48** | **10,808** | **66.5%** | **0.47** | **9,333** | **70.0%** | **0.46** | **7,813** | **73.9%** | **0.44** | **5,185** | **74.5%** | **0.44** | **<0.001** |

^a^Reported p-values are from Kruskal-Wallis equality of populations test of differences in means across years.

NE=Northeast, MW=Midwest

**eFigure 2. Logistic Model Results of Receiving Breast Conserving Surgery versus Mastectomy (n=53,060)^a,b^**

^a^p-values for ZIP3-level variables are based on clustered standard errors.

^b^Reference categories include: Year=2012; Age<50; Northeast Region; PPO/POS/Comprehensive Health Plan Type; Percent White.

**eTable 2. Trends in Median Time (in Days) to Breast Conserving Surgery (N=36,270)^a^**

| **Region** |  | **2012** | | | | | **2013** | | | | | | **2014** | | | | | |
| --- | --- | --- | --- | --- | --- | --- | --- | --- | --- | --- | --- | --- | --- | --- | --- | --- | --- | --- |
|  | **n** | **median** | **25^th^%** | **75^th^%** | **mean** | **sd** | **n** | **median** | **25^th^%** | **75^th^%** | **mean** | **sd** | **n** | **median** | **25^th^%** | **75^th^%** | **mean** | **sd** |
| NE^b^ | 936 | 22.00 | 11.00 | 35.00 | 25.39 | 20.76 | 1,833 | 22.00 | 13.00 | 35.00 | 26.19 | 21.21 | 1,610 | 24.00 | 14.00 | 36.00 | 27.20 | 21.10 |
| MW^c^ | 1,077 | 21.00 | 11.00 | 33.00 | 24.25 | 19.98 | 2,162 | 21.00 | 13.00 | 34.00 | 24.78 | 19.37 | 1,804 | 22.50 | 14.00 | 34.00 | 25.34 | 19.53 |
| South^c^ | 1,573 | 20.00 | 10.00 | 32.00 | 23.37 | 21.41 | 2,862 | 20.00 | 10.00 | 32.00 | 23.47 | 21.09 | 2,699 | 21.00 | 11.00 | 33.00 | 24.18 | 20.79 |
| West^c^ | 929 | 20.00 | 11.00 | 32.00 | 24.06 | 19.64 | 1,543 | 22.00 | 12.00 | 35.00 | 26.67 | 22.85 | 1,073 | 25.00 | 15.00 | 37.00 | 28.46 | 23.12 |
| **Overall**^c^ | **4,515** | **21.00** | **11.00** | **33.00** | **24.14** | **20.59** | **8,400** | **21.00** | **12.00** | **34.00** | **24.99** | **21.06** | **7,186** | **22.00** | **13.00** | **35.00** | **25.79** | **20.98** |
|  | | | | | | | | | | | | | | | | | | |
| **Region** |  | **2015** | | | | | **2016** | | | | | | **2017** | | | | | |
|  | **n** | **median** | **25^th^%** | **75^th^%** | **mean** | **sd** | **n** | **median** | **25^th^%** | **75^th^%** | **mean** | **sd** | **n** | **median** | **25^th^%** | **75^th^%** | **mean** | **sd** |
| NE^b^ | 1,373 | 24.00 | 13.00 | 26.00 | 27.63 | 21.73 | 1,150 | 24.00 | 14.00 | 36.00 | 27.46 | 20.98 | 846 | 24.00 | 14.00 | 36.00 | 27.19 | 20.66 |
| MW^c^ | 1,765 | 23.00 | 14.00 | 35.00 | 25.99 | 18.65 | 1,474 | 24.00 | 14.00 | 36.00 | 27.43 | 19.77 | 876 | 26.00 | 16.00 | 37.00 | 28.44 | 21.37 |
| South^c^ | 2,499 | 21.00 | 11.00 | 34.00 | 24.89 | 21.06 | 2,355 | 22.00 | 12.00 | 35.00 | 25.85 | 20.61 | 1,608 | 23.00 | 14.00 | 35.00 | 26.57 | 20.24 |
| West^c^ | 892 | 25.00 | 14.00 | 38.00 | 28.42 | 22.36 | 798 | 26.00 | 14.00 | 39.00 | 28.38 | 20.82 | 533 | 28.00 | 15.00 | 42.00 | 32.00 | 23.21 |
| **Overall**^c^ | **6,529** | **23.00** | **13.00** | **35.00** | **26.25** | **20.81** | **5,777** | **23.00** | **13.00** | **36.00** | **26.93** | **20.52** | **3,863** | **25.00** | **14.00** | **36.00** | **27.88** | **21.09** |

^a^Reported p-values are from Kruskal-Wallis equality of populations test of differences in means across years, and bivariate quantile regression with year as the only covariate (i.e., difference in medians test).

^b^Difference in Medians Test p=0.003; Difference in Means Test p=0.002

^c^Difference in Medians and Means Tests p<0.001

NE=Northeast, MW=Midwest

**eTable 3. Trends in Median Time (in Days) to Mastectomy (N=16,790)^a^**

| **Region** |  | **2012** | | | | | **2013** | | | | | | **2014** | | | | | |
| --- | --- | --- | --- | --- | --- | --- | --- | --- | --- | --- | --- | --- | --- | --- | --- | --- | --- | --- |
|  | **n** | **median** | **25^th^%** | **75^th^%** | **mean** | **sd** | **n** | **median** | **25^th^%** | **75^th^%** | **mean** | **sd** | **n** | **median** | **25^th^%** | **75^th^%** | **mean** | **sd** |
| NE^b^ | 445 | 37.00 | 22.00 | 55.00 | 42.89 | 29.30 | 775 | 37.00 | 23.00 | 53.00 | 42.47 | 29.06 | 671 | 36.00 | 23.00 | 54.00 | 42.48 | 29.26 |
| MW^c^ | 547 | 29.00 | 19.00 | 45.00 | 35.14 | 26.45 | 997 | 32.00 | 20.00 | 48.00 | 37.19 | 26.88 | 833 | 34.00 | 21.00 | 50.00 | 39.48 | 29.48 |
| South^d^ | 1,055 | 30.00 | 17.00 | 47.00 | 36.31 | 28.65 | 1,891 | 31.00 | 19.00 | 47.00 | 36.28 | 27.92 | 1,617 | 32.00 | 20.00 | 46.00 | 36.16 | 26.33 |
| West^d^ | 496 | 30.00 | 17.00 | 45.50 | 34.98 | 26.18 | 800 | 33.00 | 20.00 | 49.00 | 38.55 | 29.64 | 501 | 35.00 | 21.00 | 51.00 | 39.40 | 28.19 |
| **Overall**^d^ | **2,543** | **31.00** | **19.00** | **48.00** | **36.95** | **27.96** | **4,463** | **33.00** | **20.00** | **49.00** | **37.97** | **28.29** | **3/622** | **34.00** | **21.00** | **49.00** | **38.54** | **27.98** |
|  | | | | | | | | | | | | | | | | | | |
| **Region** |  | **2015** | | | | | **2016** | | | | | | **2017** | | | | | |
|  | **n** | **median** | **25^th^%** | **75^th^%** | **mean** | **sd** | **n** | **median** | **25^th^%** | **75^th^%** | **mean** | **sd** | **n** | **median** | **25^th^%** | **75^th^%** | **mean** | **Sd** |
| NE^b^ | 459 | 37.00 | 24.00 | 55.00 | 44.07 | 30.35 | 333 | 36.00 | 21.00 | 53.00 | 40.51 | 29.46 | 178 | 36.00 | 19.00 | 55.00 | 39.25 | 27.01 |
| MW^c^ | 642 | 34.00 | 22.00 | 48.00 | 38.01 | 26.08 | 421 | 34.00 | 22.00 | 49.00 | 37.14 | 23.80 | 302 | 35.00 | 20.00 | 48.00 | 35.29 | 23.45 |
| South^d^ | 1,314 | 34.00 | 20.00 | 49.00 | 37.83 | 27.14 | 1,018 | 34.50 | 21.00 | 50.00 | 38.54 | 27.28 | 676 | 35.00 | 20.00 | 51.00 | 38.89 | 27.94 |
| West^d^ | 389 | 34.00 | 22.00 | 50.00 | 39.84 | 26.87 | 264 | 35.00 | 22.00 | 54.00 | 39.33 | 28.67 | 166 | 40.00 | 26.00 | 56.00 | 44.75 | 30.92 |
| **Overall**^d^ | **2,804** | **34.00** | **21.00** | **50.00** | **39.17** | **27.50** | **2,036** | **35.00** | **22.00** | **50.00** | **38.68** | **27.16** | **1/322** | **35.50** | **21.00** | **51.00** | **38.85** | **27.36** |

^a^Reported p-values are from Kruskal-Wallis equality of populations test of differences in means across years, and bivariate quantile regression with year as the only covariate (i.e., difference in medians test).

^b^Difference in Medians Test p=0.45; Difference in Means Test p=0.60

^c^Difference in Medians test p<0.001; Difference in Means test p=0.02

^e^Difference in Medians and Means Tests p<0.001

NE=Northeast, MW=Midwest

**eFigure 3. Quantile (Median) Regression of Days to Breast Conserving Surgery (N=36,270)^a,b^**

^a^p-values for ZIP3-level variables are based on clustered standard errors.

^b^Reference categories include: Year=2012; Age<50; Northeast Region; PPO/POS/Comprehensive Health Plan Type; Percent White.

**eFigure 4. Quantile (Median) Regression of Days to Mastectomy (N=16,790)^a,b^**

^a^p-values for ZIP3-level variables are based on clustered standard errors.

^b^Reference categories include: Year=2012; Age<50; Northeast Region; PPO/POS/Comprehensive Health Plan Type; Percent White.
